# A Universal Peptide Matrix Interactomics Approach to Disclose Motif-Dependent Protein Binding

**DOI:** 10.1016/j.mcpro.2021.100135

**Published:** 2021-08-13

**Authors:** Evelyn Ramberger, Lorena Suarez-Artiles, Daniel Perez-Hernandez, Mohamed Haji, Oliver Popp, Ulf Reimer, Achim Leutz, Gunnar Dittmar, Philipp Mertins

**Affiliations:** 1 Max Delbrück Center for Molecular Medicine in the Helmholtz Association, Berlin, Germany; 2 Quantitative Biology Unit, Luxembourg Institute of Health, Luxembourg, Luxembourg; 3 JPT Peptide Technologies GmbH, Berlin, Germany; 4 Institute of Biology, Humboldt University of Berlin, Berlin, Germany; 5 Department of Life Sciences and Medicine, University of Luxembourg, Belvaux, Luxembourg; 6 Berlin Institute of Health (BIH), Berlin, Germany; 7 German Cancer Consortium (DKTK), partner site Berlin, Germany; 8 Deutsches Zentrum für Herz-Kreislauf-Forschung e. V. (DZHK), Berlin, Germany

**Keywords:** peptide interaction screen, proteomics, short linear motifs, post-translational modifications, point mutations, ABC, ammonium bicarbonate, AGC, automated gain control, CAA, chloroacetamide, CRs, conserved regions, EGFR, epidermal growth factor receptor, FDR, false discovery rate, IDRs, intrinsically disordered regions, IT, injection time, LFQ, label-free quantification, MBR, match between run, MoRFs, molecular recognition features, PPIs, protein–protein interactions, PRISMA, protein interaction screen on a peptide matrix, PTMs, post-translational modifications, SH2, Src homology 2, SLiMs, short linear motifs, TCEP, Tris(2-carboxyethyl)phosphine hydrochloride

## Abstract

Protein–protein interactions mediated by intrinsically disordered regions are often based on short linear motifs (SLiMs). SLiMs are implicated in signal transduction and gene regulation yet remain technically laborious and notoriously challenging to study. Here, we present an optimized method for a protein interaction screen on a peptide matrix (PRISMA) in combination with quantitative MS. The protocol was benchmarked with previously described SLiM-based protein–protein interactions using peptides derived from EGFR, SOS1, GLUT1, and CEBPB and extended to map binding partners of kinase activation loops. The detailed protocol provides practical considerations for setting up a PRISMA screen and subsequently implementing PRISMA on a liquid-handling robotic platform as a cost-effective high-throughput method. Optimized PRISMA can be universally applied to systematically study SLiM-based interactions and associated post-translational modifications or mutations to advance our understanding of the largely uncharacterized interactomes of intrinsically disordered protein regions.

## Highlights

• Optimized protocol for analysis of peptide–protein interactions with peptide arrays.
• Detection of interactions affected by mutations or post-translational modifications.
• Mapping of interaction sites with overlapping peptide sequences.
• Implementation on a liquid-handling robotic platform.

## Graphical Abstract

**Figure:**
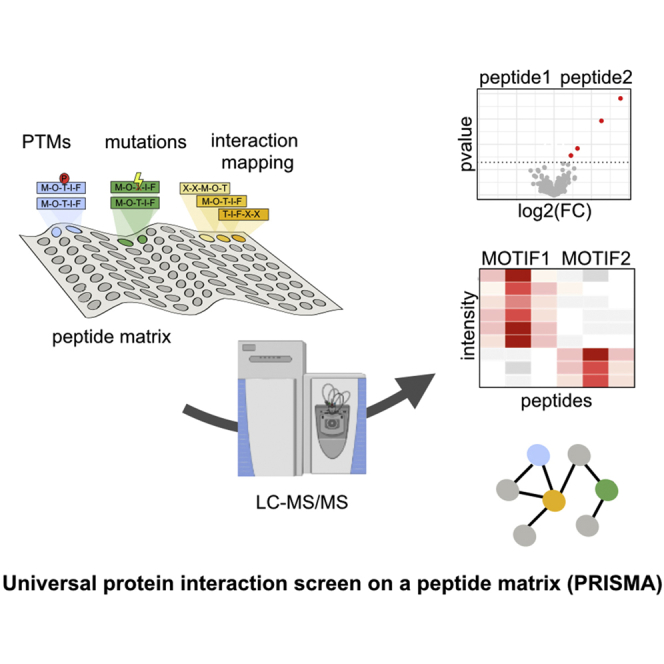

## In Brief

A universal and optimized peptide matrix pull-down approach enables cost-effective and reproducible high-throughput interactome analysis of peptides with reduced analysis time. Our data provide proof of principle for the wide applicability of our streamlined protocol to map and detect motif-based protein interactions influenced by mutations and post-translational modifications.

Many protein–protein interactions (PPIs) are mediated by intrinsically disordered regions (IDRs). Within IDRs, short linear motifs (SLiMs, typically 3–12 amino acid) and molecular recognition features (MoRFs; up to 30 amino acids) may serve as contact points or induced fit regions, respectively, to accomplish interactions with partner proteins. More than 30% of the human proteome is at least partially intrinsically disordered, and current estimates suggest that these regions contain more than 100,000 SLiMs (1). PPIs mediated by such short, unstructured protein regions are dynamic and transient, making them instrumental to signal transduction and gene regulatory processes (1, 2, 3, 4). Mutations disrupting SLiMs and MoRFs are involved in pathological processes, and in comparative sequence analyses, it was demonstrated that within SLiM containing IDRs, disease-causing mutations are more likely to occur than neutral missense mutations (5). Additional complexity of SLiM-based interactions results from post-translational modifications (PTMs) that frequently occur within or adjacent to SLiMs/MoRFs and that modulate protein interactions (6). Such effects are challenging to study with traditional biochemical methods but can be recapitulated with peptide–protein pull-downs using synthetic peptides as baits.

Chemically synthesized SLiM peptides can be used for protein-interaction discovery and, in addition to proteinogenic amino acids, amino acids with side-chain modifications can be incorporated. This aids the detection of PTM-specific binding events and poses a clear advantage over conventional protein pull-downs. Capture of interacting proteins is facilitated by immobilizing the synthetic peptides on beads through a linker group (7, 8, 9) or by using peptide libraries directly synthesized on a solid membrane support (10).

Protein pull-downs with SPOT-synthesized peptide arrays on cellulose supports (11) provide a cost-effective and scalable tool to study protein–protein interactions mediated by short IDRs. In the past, peptide arrays have been widely used by numerous studies for epitope mapping (12, 13, 14) and mapping of protein interactions with an antibody-based approach similar to far Western blotting (15, 16). While the latter is restricted to the detection of a specific protein by an antibody, mass spectrometric analysis of the bound fraction aids the unbiased identification of all interacting proteins (10, 17, 18, 19, 20).

In the protein interaction screen on a peptide matrix (PRISMA) workflow, synthetic peptide arrays are incubated with the cell lysate and, after washing, individual peptide spots are excised and processed in a microwell format. Proteins binding to each peptide spot are subsequently identified by quantitative MS. PRISMA has been recently used to map protein interactions along the amino acid sequence and PTM sites of intrinsically disordered CEBP transcription factors and to examine how disease-causing point mutations affect protein interactions (18, 19, 20). Here, we present an optimized PRISMA method and demonstrate robustness and universal applicability of the high-throughput screening approach to map protein interactions across unstructured regions carrying PTMs or mutations.

## Experimental Procedures

### Membrane Design

Custom cellulose peptide membranes (C-terminal attachment) were ordered from JPT Peptide Technologies. Peptides were designed with a length of 14 amino acids and acetylated N termini. Peptide sequences are available in supplemental data (supplemental Table S1).

### HeLa Cell Extract Preparation

HeLa cells were cultured in 15-cm dishes in Dulbecco's modified Eagle's medium supplemented with 10% fetal bovine serum (Gibco), penicillin/streptomycin mix (Sigma), and 1 mM sodium pyruvate (PAN Biotech). Cells were washed with PBS and harvested *via* scraping in ice-cold PBS, followed by centrifugation (5 min, 1000*g* at 4 °C). Cell pellets were lysed with chilled modified RIPA buffer (18) (50 mM Hepes, pH 7.6, 150 mM NaCl, 1 mM EGTA, 1 mM MgCl_2_, 10% glycerol, 0.5% Nonidet NP-40, 0.05% SDS, 0.25% sodium deoxycholate supplemented with 2 μg/ml aprotinin, 10 μg/ml leupeptin, and 1 mM PMSF). Universal nuclease (Pierce, Thermo Fisher) was added, samples were vortexed for 2 s, and cell lysates were incubated on ice for 30 min followed by a second vortex pulse and centrifugation at high speed (10 min, 17,000*g*, 4 °C) to remove cell debris. Lysates were snap-frozen and stored at −80 °C until use. The commercial HeLa nuclear extract (20 mM Hepes pH 7.9, 100 mM KCl, 0.2 mM EDTA, 20% Glycerol; (21)) was obtained from Ipracell. Before experiments, extracts were thawed on ice, centrifuged at high speed, and the supernatant was diluted to the desired concentration.

### PRISMA Protocol Optimization

PRISMA experimental conditions were optimized stepwise to determine optimal parameters. As a starting point, a protocol adapted from the workflows described by Meyer *et al*. (18) and Dittmar *et al*. (19) was used: In brief, peptide membranes were conditioned with the PRISMA washing buffer (50 mM Hepes, pH 7.6, 150 mM NaCl, 1 mM EGTA, 1 mM MgCl_2_, 10% glycerol), followed by incubation with the RIPA cell lysate (45 min, shaking, 4 °C), and three washing steps, 5 min each with the PRISMA washing buffer. After air-drying of membranes, individual peptide spots were punched out with a 2-mm biopsy puncher (Stiefel) and placed into a 96-well plate containing 20 μl of the denaturation buffer (6 M urea, 2 M thiourea, 10 mM Hepes, pH 8) per well. After in-solution digestion and desalting *via* STAGE (22) tips, samples were subjected to LC-MS/MS analysis. We optimized this default protocol by modification of one parameter at a time, using three epidermal growth factor receptor (EGFR) peptides in technical quadruplicates or triplicates as a reference. Protein concentration was tested at 1, 2, 4, and 8 mg/ml while keeping the incubation time at 45 min. In a second experiment, protein concentration was kept at 4 mg/ml and incubation times were tested at 10, 20, 45, and 90 min. Influence of temperature during washing (at 4 °C and at room temperature [RT]) was also evaluated as an indicator of the suitability of the protocol for automation. Different HPLC gradient lengths were tested by modifying the length of the linear part of the gradient (10, 20, or 45 min) as described. All other samples were analyzed with a 20-min gradient (total method length 40 min). Results of optimization experiments are available in supplemental Table S2–S5.

### Universal PRISMA Method

All incubation and washing steps were performed on an orbital shaker at 4 °C in a plastic box. PRISMA membranes were preconditioned with the PRISMA washing buffer for 15 min. After preconditioning, the membrane was incubated with the HeLa cell lysate (4 mg/ml) for 20 min on ice and washed three times for 5 min each with the cold PRISMA washing buffer. After washing, the membrane was placed on a glass plate and air-dried (~1 h). After drying, each spot was punched out with a 2-mm biopsy puncher and fully immersed in 20-μl denaturation buffer in a 96-well plate. At this point, samples can be frozen at −80 °C before continuing with in-solution digestion. A more detailed, stepwise protocol for PRISMA is available in supplemental data.

### In-Solution Digestion of PRISMA samples

For in-solution digestion, samples were first reduced by with 2 μl of 10 mM Tris(2-carboxyethyl)phosphine hydrochloride (TCEP) for 30 min, followed by alkylation with 2 μl of 55 mM chloroacetamide (CAA) for 45 min. Samples were diluted with 100 μl of 50 mM ammonium bicarbonate (ABC) and digested overnight by adding 0.5 μg of sequencing-grade trypsin (Promega) and 0.5 mAU of sequencing-grade LysC (Wako) per sample. Digested samples were acidified with 4 μl of 25% TFA and desalted with STAGE tips as described previously (22).

### MS Data Acquisition

Desalted and dried samples were resuspended in 12 μl MS sample buffer (3% acetonitrile/0.1% formic acid v/v), and 2 μl of the sample was separated online with an Easy-nLC 1200 system directly coupled to a Q Exactive HF-X mass spectrometer (both Thermo Fisher Scientific). Samples were separated on a 20-cm reverse-phase column (inner diameter 75 μm, packed in-house with 3-μm C18 Reprosil beads) at a flow rate of 250 nl/min at 55 °C. For testing the optimal gradient length, the gradient was ramping from 3% to 81% acetonitrile in 10, 20, or 45 min, followed by a plateau at 81% acetonitrile for 2 min and 45% acetonitrile for 10 min (total gradient length 32 min). For all other samples, the linear gradient part was set to 20 min. MS data were acquired in a positive data-dependent acquisition mode with a top20 method. Full-scan MS spectra were acquired at a resolution of 60,000 in the scan range from 350 to 1800 m/z, automated gain control (AGC) target value of 3e6, and maximum injection time (IT) of 10 ms. MS/MS spectra were acquired at a resolution of 15,000, AGC target of 1e5, and maximum IT of 25 ms. Ions were isolated with a 1.3 m/z isolation window, and normalized collision energy was set to 28. Unassigned charge states and charge states 1, 7, or higher were excluded from fragmentation. The minimum AGC target was set to 5e3, and dynamic exclusion was set to 20 s. The acquisition queue was randomized, and blank injections were run every fourth sample. The MS proteomics data and search results have been deposited to the ProteomeXchange Consortium *via* the PRIDE partner repository (23) with the dataset identifier PXD027265.

### Evosep Sample Processing and MS Data Acquisition

Digested PRISMA peptides were desalted with Evotips (Evosep) according to the manufacturer's instructions. In brief, Evotips were conditioned with 20-μl solvent B (0.1% formic acid in acetonitrile), followed by soaking in 1-propanol and washing with 20-μl solvent A (0.1% formic acid in water). Samples were acidified, and 20% of one PRISMA sample was loaded in 20 μl 1% formic acid (v/v) on one Evotip. Evotips were washed once with 20-μl solvent A. Samples were separated online with the Evosep LC system choosing the preset 60 samples/day gradient (21 min). The Evosep system was directly coupled to a Q Exactive HF-X mass spectrometer set to the positive data-dependent acquisition mode with a top20 method. Full-scan MS spectra were acquired at a resolution of 60,000 in the scan range from 350 to 1800 m/z, AGC target value of 3e^6^, and maximum IT of 10 ms. MS/MS spectra were acquired at a resolution of 15,000, AGC target of 1e^5^, and maximum IT of 22 ms. Ions were isolated with a 1.3 m/z isolation window and normalized collision energy was set to 28. Unassigned charge states and charge states 1, 7, or higher were excluded from fragmentation. The minimum AGC target was set to 1.1e^3^, and dynamic exclusion was set to 20 s.

### Automation on Bravo

PRISMA spots were punched out from the dry membrane and placed individually in the wells of a 96-well plate. Incubation with the protein extract, washing, digestion, and desalting were performed on an AssayMAP Bravo liquid-handling platform (Agilent). For automation, the whole deck of an AssayMAP Bravo workbench was used and included seven individual plate positions plus the tip-parking station and the tip-washing station. One position was equipped with a shaking tool, and an additional cooling unit allowed to keep the cell lysate at 4 °C. For transfer of liquids, an LT-head equipped with 250-μl tips was installed. Three deck positions were allocated with stacked plates, allowing an overall number of nine plates to be handled. The nine plates included LysC/trypsin mixture (0.5 μg of trypsin and 0.5 mAU of LysC), TFA solution (10% v/v), cell lysate at 4 °C, 10 mM TCEP solution, 55 mM CAA solution, PRISMA washing buffer, denaturation buffer, 50 mM ABC buffer, and the sample plate. Before each transfer step, tips were washed using the tip-washing station. After each transfer step, the liquid was pipetted up and down thrice. At first, 200 μl of the washing buffer was added to the wells containing the membranes and shaken for 15 min. The buffer was subsequently removed and replaced by 50 μl of the cell lysate and incubated for 20 min while shaking briefly in 1-min intervals. After removing the cell lysate, samples were washed using 200-μl washing buffer and incubated for 5 min while shaking. The washing step was repeated twice. The buffer was removed and replaced by 20-μl denaturation buffer, followed by 2-μl TCEP solution and incubated for 30 min while shaking. A volume of 2-μl CAA solution was added and incubated 45 min while shaking. Samples were then diluted using 100 μl ABC buffer, followed by the addition of 4 μl LysC/trypsin mixture and shaken for 16 h. The reaction was stopped by adding 5-μl TFA solution. Peptides were desalted using the standard AssayMAP peptide cleanup protocol with C18 cartridges. Desalted peptides were analyzed with LC-MS/MS as described.

### Raw Data Search

Raw files were analyzed using MaxQuant, version 1.5.2.8, searching against the human UniProt database (2017) containing reviewed entries, isoforms, and unreviewed entries (total 92,931 entries) and common contaminants. Protease specificity was set to trypsin. A maximum of two missed cleavage sites per peptide were allowed. Fixed modifications were set to carbamidomethylation of C and variable modifications were set to M oxidation, N-terminal acetylation (M), and deamidation (NQ). A maximum of five modifications per peptide were allowed. N-terminal acetylation and M-oxidation were used in protein quantification (unmodified counterpart discarded). The minimal peptide length was set to seven amino acids, and the main search peptide tolerance was set to 4.5 ppm. Modified peptide identifications with a score greater than 40 and a delta score greater than 6 were allowed. The false discovery rate (FDR) was set to 0.01 on protein and peptide level. Minimal ratio count was set to 2. For all runs, the fast label-free quantification (fast LFQ) was enabled. The match between run (MBR) option was disabled unless otherwise stated. Search results (protein groups and peptide identifications) have been deposited to the ProteomeXchange Consortium *via* the dataset identifier mentioned above.

### Experimental Design and Statistical Rationale

Each PRISMA peptide sequence was analyzed in quadruplicates (four separate membrane spots). LFQ data were imputed and analyzed with a two-sample *t* test, similar to the analysis strategy of other label-free large-scale affinity purification MS data (24, 25). Analysis of text output files from MaxQuant was performed using the R software environment. Protein groups were filtered for contaminants, reverse hits, and proteins identified with less than three peptides or only identified by site. After filtering for at least three valid values per group, missing values were imputed separately for each group as described previously (24). LFQ intensities of modified peptides were compared with their unmodified counterpart by pairwise comparisons with a moderated two-sample *t* test from the limma package (26). *p*-values were corrected with the Benjamini–Hochberg method, and an FDR cut-off < 0.05 was implemented. For the analysis of SLIM interactions, a similar approach was used. All peptides originating from the same region of a protein (e.g., CR2 of CEBP) were compared against each other in a pairwise manner with a moderated two-sample *t* test. In addition to the FDR cut-off (<0.05), interacting proteins were filtered based on intensity profiles across overlapping peptide sequences as described before (19). An interaction was considered a true interaction if an LFQ signal was detected also in overlapping PRISMA peptides sharing more than 50% sequence overlap. Processed search results are available in supplemental data.

## Results

### Optimization of Experimental Conditions

**Fig. 1:**
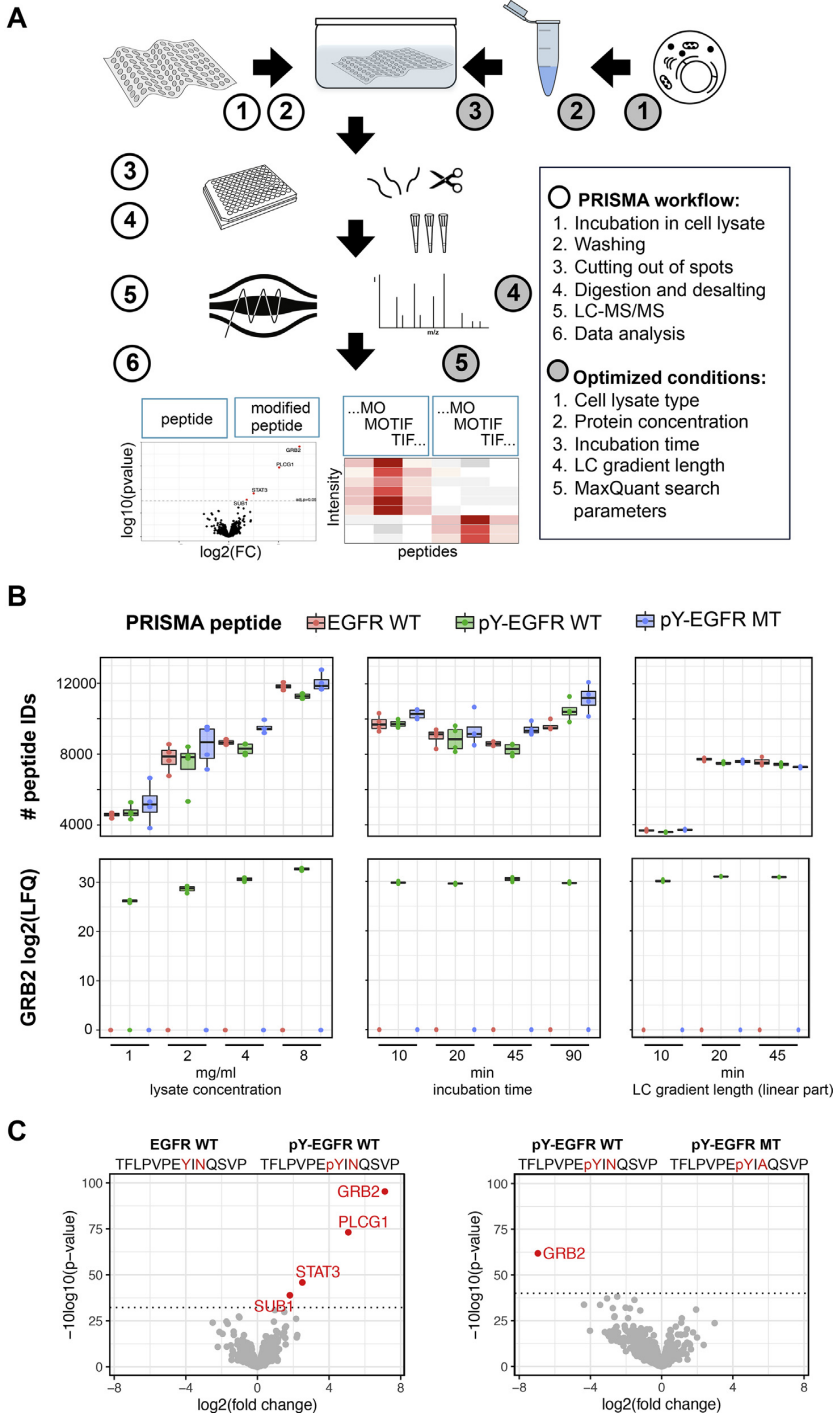
**Optimization of experimental conditions for PRISMA**. *A*, schematic representation of the PRISMA workflow and conditions optimized in the protocol. *B*, number of identified peptides and GRB2 LFQ intensity across EGFR WT, EGFR phosphorylated (pY1092) and EGFR phosphorylated mutated (pY1092, N1094>A) peptides dependent on protein lysate concentration (n = 4), incubation time (n = 4), and LC gradient length (n = 3). Box plots display medians of nonzero measurements. Data from optimization experiments are available in supplemental Tables S2–S5. *C*, results of EGFR PRISMA pull-downs with optimal conditions. Quadruplicates of PRISMA peptide pull-downs were analyzed with label-free LC-MS/MS, and pairwise comparisons were performed with a moderated two-sample *t* test. Volcano plots depict fold changes of detected proteins plotted against their *p*-value. Significance cut-off (0.05 FDR) is indicated with a *dotted line*. Processed data are available in supplemental Table S6. FDR, false discovery rate; LFQ, label-free quantification; PRISMA, protein interaction screen on a peptide matrix.

To provide a universal workflow and a streamlined protocol for various applications, we first optimized experimental PRISMA conditions using prototypic peptides derived from the EGFR as a well-described reference. The adaptor protein GRB2 contains an Src homology 2 (SH2) domain that facilitates interactions with phosphorylated tyrosine residues in the C terminus of the EGFR. A tyrosine phosphorylated (Y1092) EGFR peptide has been previously shown to interact with GRB2 in bead-based peptide protein pull-downs, and the interaction was disrupted by a mutation two amino acids downstream the phosphorylation site (N1094A) (8). We recapitulated these findings in a PRISMA-type setting, using peptides immobilized on cellulose membranes. The experimental procedure was optimized stepwise by modifying several experimental parameters, including protein lysate concentration, incubation time, LC gradient, and washing conditions as described in detail in the Experimental Procedures section (Fig. 1*A*).

First, we examined the effect of the protein lysate concentration on the number of identified peptides, as a proxy for background binding *versus* the specific GRB2 LFQ signal of the EGFR–pY1092 peptide. The excellent reproducibility and high signal-to-noise ratio of GRB2 in EGFR pull-downs allowed for robust assessment of the influence of experimental parameters in PRISMA. Except for the experiment with 1 mg/ml protein lysate, GRB2 was identified in all replicates of the PRISMA pull-downs with phosphorylated EGFR peptides but in none of the other EGFR pull-downs. We also observed less variance in GRB2 LFQ signal intensities with higher protein lysate concentrations. A protein lysate concentration of 8 mg/ml resulted in the strongest GRB2 LFQ signal, although a protein concentration of 4 mg/ml was sufficient to obtain strong signals with a good signal-to-noise ratio and was easier to generate and to handle (Fig. 1*B*, supplemental Table S2). Previous PRISMA studies used incubation times between 30 and 120 min. As shown in Figure 1*B*, variation of incubation time had little effect on the number of peptide identifications or GRB2 LFQ intensity, demonstrating the robustness of the method (supplemental Table S3). As the best practice condition, we decided on an incubation time of 20 min, as this simplified reproducible handling while keeping the overall protocol relatively short. As expected for a low-complexity proteome sample, modifying LC-gradient length affected the amount of total identified peptides and proteins but not the GRB2 LFQ signal (Fig. 1*B*, supplemental Table S4). We observed no additional gain in peptide identifications beyond a gradient length of 20 min (total MS method length 40 min including overhead time) although enabling the MBR option of MaxQuant may recover additional low-abundant features in samples analyzed with longer gradients. In addition to monitoring the GRB2 signal, which represents a best-case scenario example, we also extracted the LFQ intensity of two other SH2 domain containing EGFR interactors (STAT3, PLCG1) in the optimization experiments (supplemental Fig. S1*A*, supplemental Table S2–S5). Although less pronounced and more variable than GRB2, the LFQ intensity of STAT3 and PLCG1 is also increasing with higher lysate concentrations. Incubation time and gradient length did not have an effect on signal strength. Other than GRB2 and PLCG1, STAT3 is also detected in nonphosphorylated EGFR PRISMA pull-downs and the interaction of STAT3 with pY-EGFR is not abrogated by the N1094A point mutation downstream the EGFR phosphorylation site.

Because constant cooling is difficult to implement on some robotic systems during an automated workflow, we also tested the effect of different washing temperatures on PRISMA results in a direct comparison. While we recommend washing samples at 4 °C, the differential interaction of GRB2 was also identified with washing at RT, resulting in slightly lower, yet acceptable LFQ signal intensities (supplemental Fig. S1*B*, supplemental Table S5). In accordance with these results, all following PRISMA experiments were performed with 4 mg/ml protein lysate at 4 °C, 20 min incubation time, washing at 4 °C and analyzed with a 20 min LC gradient. Using these experimental conditions, we detected 1145 proteins (9763 peptides) in EGFR PRISMA pull-downs of which 4 proteins (GRB2, PLCG1, STAT3, and SUB1) differentially interacted (<0.05 FDR) with the tyrosine-phosphorylated peptide compared with the nonmodified counterpart (Fig. 1*C*).

### Applications of PRISMA

Next, we applied the optimized PRISMA workflow to peptide–protein interactions falling into three different categories: (1) interactions affected by amino acid variations in the bait sequence, (2) interactions affected by PTMs, and (3) fine mapping of SLiM-mediated protein interaction sites by tiling peptides. In total, we screened 27 peptides in quadruplicates derived from seven different proteins (supplemental Table S1) for protein interactions with the modified RIPA whole-cell extract (supplemental Table S6) or nuclear extract (supplemental Table S7). On average, 886 proteins (7490 peptides) were identified per peptide spot, and the average Pearson correlation between replicates was 0.91, indicating high reproducibility. These examples demonstrate the versatility of PRISMA and provide guidance for common MS data analysis options and positive controls in prospective PRISMA screens using different applications.

### Point Mutations Within Protein Interaction Motifs

**Fig. 2:**
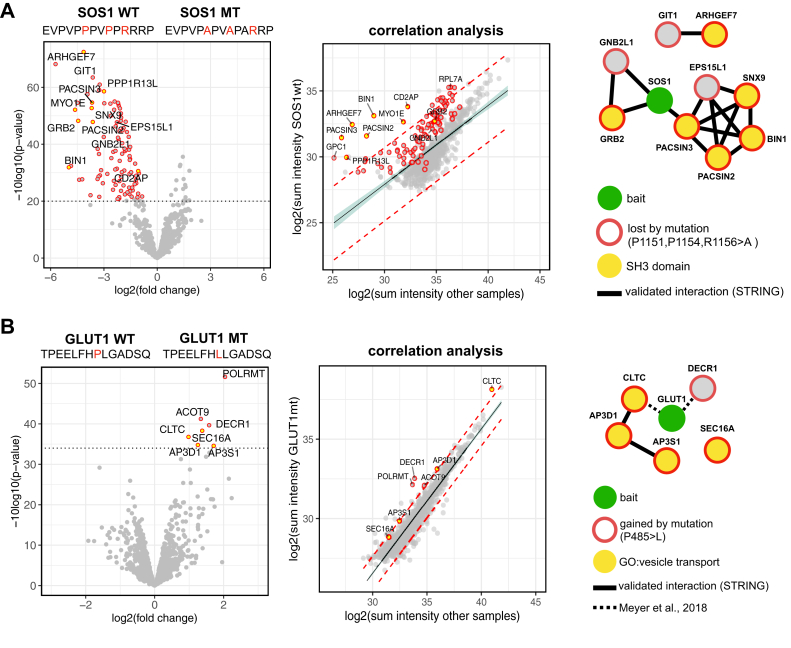
**PRISMA detects gain and loss of protein binding to mutant peptides.***A*, mutation of an SOS1 peptide disrupts the SH3-binding motif and causes loss of interactors. *B*, the disease-causing point mutation in GLUT1 (P485L) creates a dileucine motif that interacts with clathrin and endocytosis adaptor proteins as previously described by Meyer *et al*., (18). *A* and *B*, quadruplicates of PRISMA peptide pull-downs were analyzed with label-free LC-MS/MS. WT and mutated (MT) peptide sequences were compared with a moderated two-sample *t* test. Volcano plots depict fold changes of detected proteins plotted against their *p*-value. Significance cut-off (0.05 FDR) is indicated with a *dotted line*. Correlation plots show log2 (summed intensities) of proteins across peptide spots. Linear regression line (*black line*) with 95% confidence interval and 95% prediction interval (*dotted red line*) is indicated. SOS1 interactors detected only in SOS1 WT peptides (SNX9, GIT1, EPS15L1) were removed from the correlation plot in *A*. Interaction networks display subset of significant interactors connected to the bait in a STRING network or with the GO annotation vesicle transport (*A*) or SH3 domain (*B*). Processed data are available in supplemental Table S6. FDR, false discovery rate; PRISMA, protein interaction screen on a peptide matrix.

Amino acid sequence alterations may lead to the gain or loss of protein interaction motifs. The guanine exchange factor SOS1 contains a polyproline type II motif PX⌽PXR that binds to SH3 protein-interaction domains (27). Consequently, disruption of this proline motif leads to the loss of SH3 domain-interaction patterns (8). We included an SOS1 peptide spanning the proline motif and a mutated version (P1151, P1154, R1156>A) in the PRISMA setup. The mutation and disruption of the proline motif led to the loss of 100 interaction partners, including nine proteins with SH3 domains among the most differential interactors (Fig. 2*A*, supplemental Table S6). The other, non-SH3 domain containing proteins may represent indirect interactors of SOS1 within a protein complex or nonspecific background binders of the WT peptide sequence. To further discriminate high confidence interactors, we performed correlation analysis of protein intensities in the WT SOS1 PRISMA pull-down against the PRISMA background (summed intensity per protein across other peptide pull-downs). Calculation of the 95% prediction interval of a linear regression highlights 24 proteins with an enhanced signal-to-noise ratio in SOS1 PRISMA pull-downs as well as three interactors (SNX9, GIT1, and EPS15L1) that were exclusively detected in the SOS1 PRISMA pull-down. This indicates that these proteins represent highly specific direct or indirect, protein complex–based interactors of the WT SOS1 peptide that are lost upon mutation of the peptide sequence. The other proteins, albeit significant in a direct SOS1 WT mutant comparison, include nonspecific background binders to the SOS1 WT peptide that were detected with lower intensity in the mutant version.

A single point mutation (P485L) in the disordered cytosolic region of the glucose transporter GLUT1 creates an endocytosis motif that leads to aberrant clathrin-mediated endocytosis of the protein and causes GLUT1 deficiency syndrome (18). Here, we identified seven differential interactors of the mutated GLUT1 peptide over the WT peptide, including two mutation-specific interactors previously identified by Meyer *et al*. (18) (CLTC and DECR1) and three additional proteins involved in endocytosis and vesicle transport (SEC16A, AP3D1, and AP3S1) (Fig. 2*B*, supplemental Table S6). Differences in the identified interactors may be due to different cell lines, quantification techniques, and mass spectrometers used in both studies. Correlation analysis of protein intensities in the mutant GLUT1 peptides against the PRISMA background highlights CLTC, DECR1, POLRMT, and AP3D1 as the most confident differential interactors.

The cytoskeletal WASP-interacting protein was previously identified to interact with the SH3 and SH2 domains containing adaptor protein NCK1 (28) *via* a polyproline type II motif (29). We included a WASP-interacting protein peptide containing the motif and a mutated peptide (P332, P335>A) in our PRISMA setup (supplemental Fig. S2*A*). The differential interaction of NCK1 was only identified after enabling the MBR option in the MaxQuant proteomics software (30). The MBR option transfers identifications of MS1 features between LC-MS/MS runs or samples based on retention time and precursor mass values, thereby increasing not only the overall sensitivity but also the overall background in identifications per sample. For the analysis of GLUT1 and SOS1 peptides, enabling the MBR option concomitantly increased the number of interactors that were not functionally connected to the expected differential interactor (supplemental Fig. S2*B*). Similarly, MBR was able to recover GRB2 identification in pY-EGFR PRISMA samples prepared with low-input lysate concentrations but also increased the background signal in other samples as well as variability (supplemental Fig. S2*C*). We therefore recommend to analyze PRISMA data initially without using MBR but to consider this option after inspecting the results.

### PTMs Within Protein Interaction Motifs

**Fig. 3:**
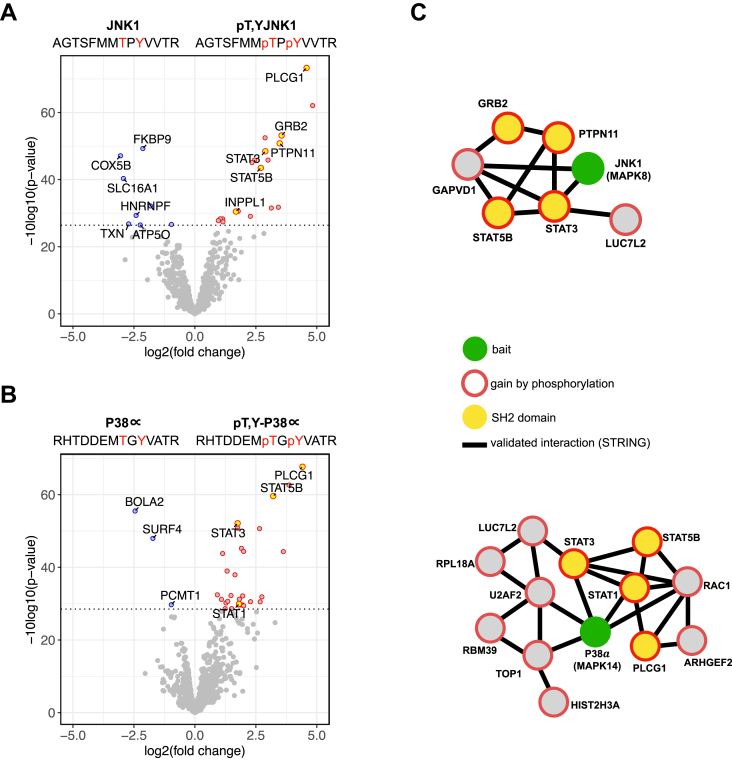
**PRISMA identifies phosphorylation-dependent interactions of kinase activation loop peptides**. Quadruplicates of PRISMA peptide pull-downs were analyzed with label-free LC-MS/MS. Pairwise comparison of unmodified and phosphorylated JNK1 (*A*) or P38α (*B*) peptides was performed with a moderated two-sample *t* test. Volcano plots depict fold changes of detected proteins plotted against their *p*-value. Significance cut-off (0.05 FDR) is indicated by a *dotted line*. *C*, STRING protein interaction networks display interactors gained by phosphorylation and connected to the bait with less than three edges. Processed data are available in supplemental Table S6. FDR, false discovery rate; PRISMA, protein interaction screen on a peptide matrix.

PTMs are frequently located within IDRs and function as regulators of SLiM-based protein interactions. We analyzed phosphorylated and unmodified peptides derived from the activation loop of MAP kinase JNK1 (MAPK8) and MAP kinase p38 alpha (MAPK14) that both play an important role in signaling processes regulating cell proliferation, survival, and differentiation (31). PRISMA identified 20 (JNK1, Fig. 3*A*, supplemental Table S6) and 29 (p38α, Fig. 3*B*, supplemental Table S6) proteins differentially interacting with the phosphorylated peptides, as compared with their unmodified counterpart. In total, 7 of the phosphorylation-specific interactors of JNK1 and p38α identified by PRISMA contained SH2 domains (STAT5B, STAT3, STAT1, PLCG1, GRB2, PTPN11, INPPL1; Fig. 3*C*), and we observed differential binding patterns of individual proteins. Most interestingly, we found STAT1 as a differential interactor of the phosphorylated p38α (FDR 0.04; FC 1.8) but not the JNK1 (FDR 0.23; FC 0.7) peptide in concordance with the previous finding that p38 kinase activity is required for the activating phosphorylation of STAT1 at S727 (32). Our results thus provide evidence for a feedback mechanism involving an activating phosphorylation event on p38 for its binding to STAT1.

Substrates of JNK1 and p38 include both cytoplasmic and nuclear proteins. To explore the effects of different cell lysis and protein extraction approaches, we performed PRISMA pull-downs with modified RIPA whole-cell extracts and commercial nuclear extract (supplemental Table S7) and found partially overlapping interaction partners, including proteins with SH2 domains (supplemental Fig. S3). As expected, pull-downs with total-cell lysates and modified RIPA buffer extraction showed a broader range of interactors, as compared with a more discrete list of interactors contained in nuclear extracts.

### Mapping of Protein-Interaction Motifs

**Fig. 4:**
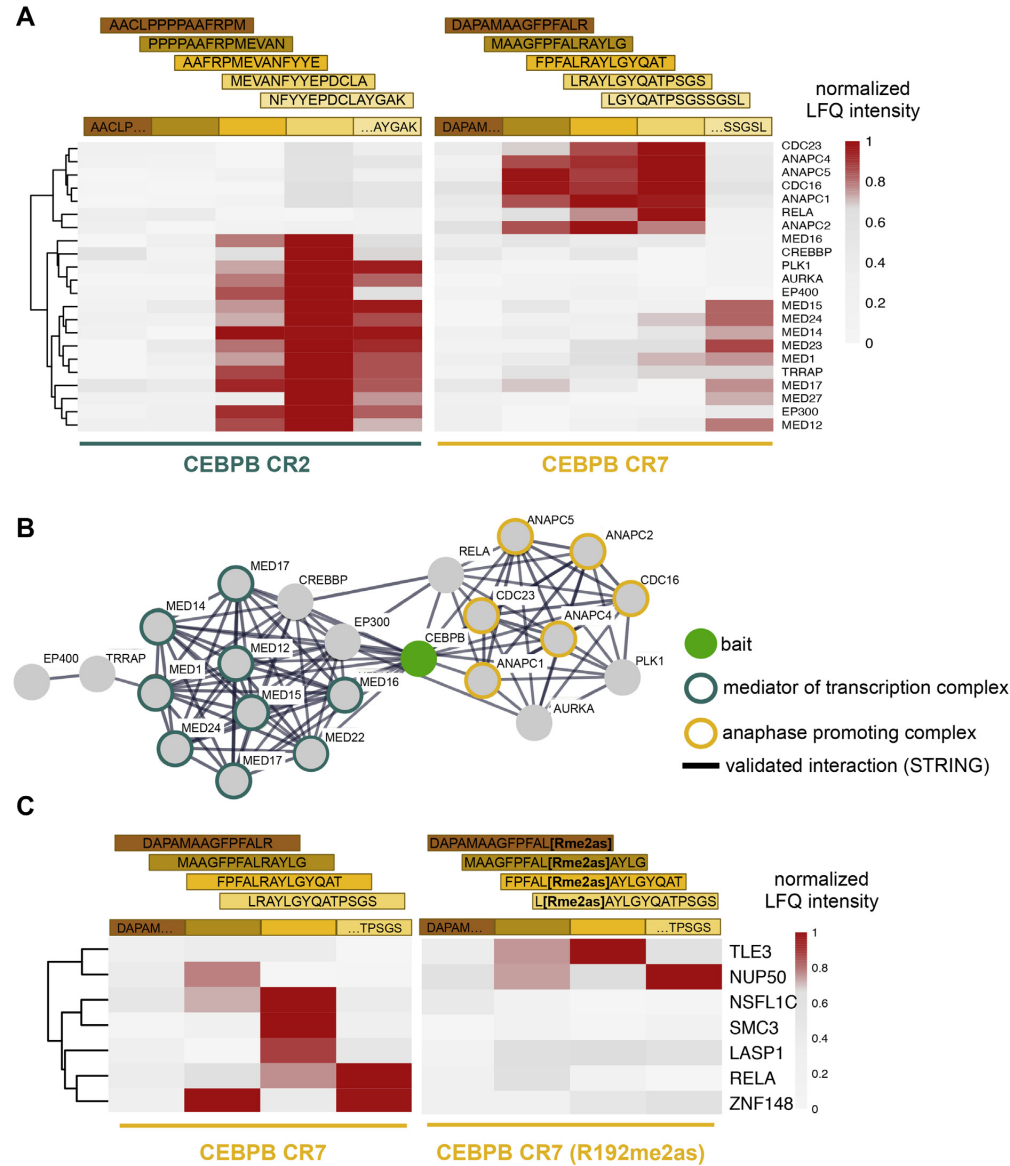
**PRISMA maps SLiM- and methylation-dependent protein interactions of CEBPB**. CEBPB peptides covering conserved regions CR2 and CR7 were screened for protein interactions with PRISMA. Heat map color scales represent normalized LFQ intensities across PRISMA peptides. *A*, extracted PRISMA-binding profiles of significant interactors in CR2 and CR7 overlapping with Dittmar *et al.* (19). *B*, STRING protein interaction network of CEBPB CR2/CR7 interactors in panel A. Members of the mediator of transcription (mediator) complex and the anaphase-promoting complex/cyclosome (APC/C) are highlighted. *C*, extracted binding profiles of interactions regulated by CEBPB R193 methylation. Processed data are available in supplemental Table S7. CRs, conserved regions; LFQ, label-free quantification; PRISMA, protein interaction screen on a peptide matrix; SLiMs, short linear motifs.

PRISMA can be used to identify and fine-map protein-interaction motifs, such as SLiMs and MoRFs (16, 19). Here, we performed PRISMA with peptides covering the conserved regions (CRs) CR2 and CR7 of the transcription factor CEBPB using commercial nuclear HeLa extracts (33). Peptides were designed with a sequence overlap of four amino acids, and significant interactors were determined by a *t* test-based approach, as described in the Experimental Procedures section. Inclusion of peptides with overlapping sequences allowed for an additional filtering step based on the intensity profile of interacting proteins across neighboring peptide sequences, as described previously (19). Components of the mediator of transcription (mediator) and anaphase-promoting complex bind to CEBPB CR2 and CR7, respectively, and the binding patterns clearly showed peaks at the center of the regions with some residual binding in neighboring peptides (Fig. 4, *A* and *B*, supplemental Table S7). In a principal component analysis, PRISMA CEBPB samples clustered together by the CR origin and protein complex binding (supplemental Fig. S4). In addition, we confirmed the R193 methylation-dependent interactions of CEBPB CR7 with TLE3, NUP50, NSFL1C, SMC3, LASP1, RELA, and ZNF148 (Fig. 4*C*) that have been previously described and validated by immunoprecipitation protein blotting analysis (19).

### Increasing the Throughput of PRISMA

**Fig. 5:**
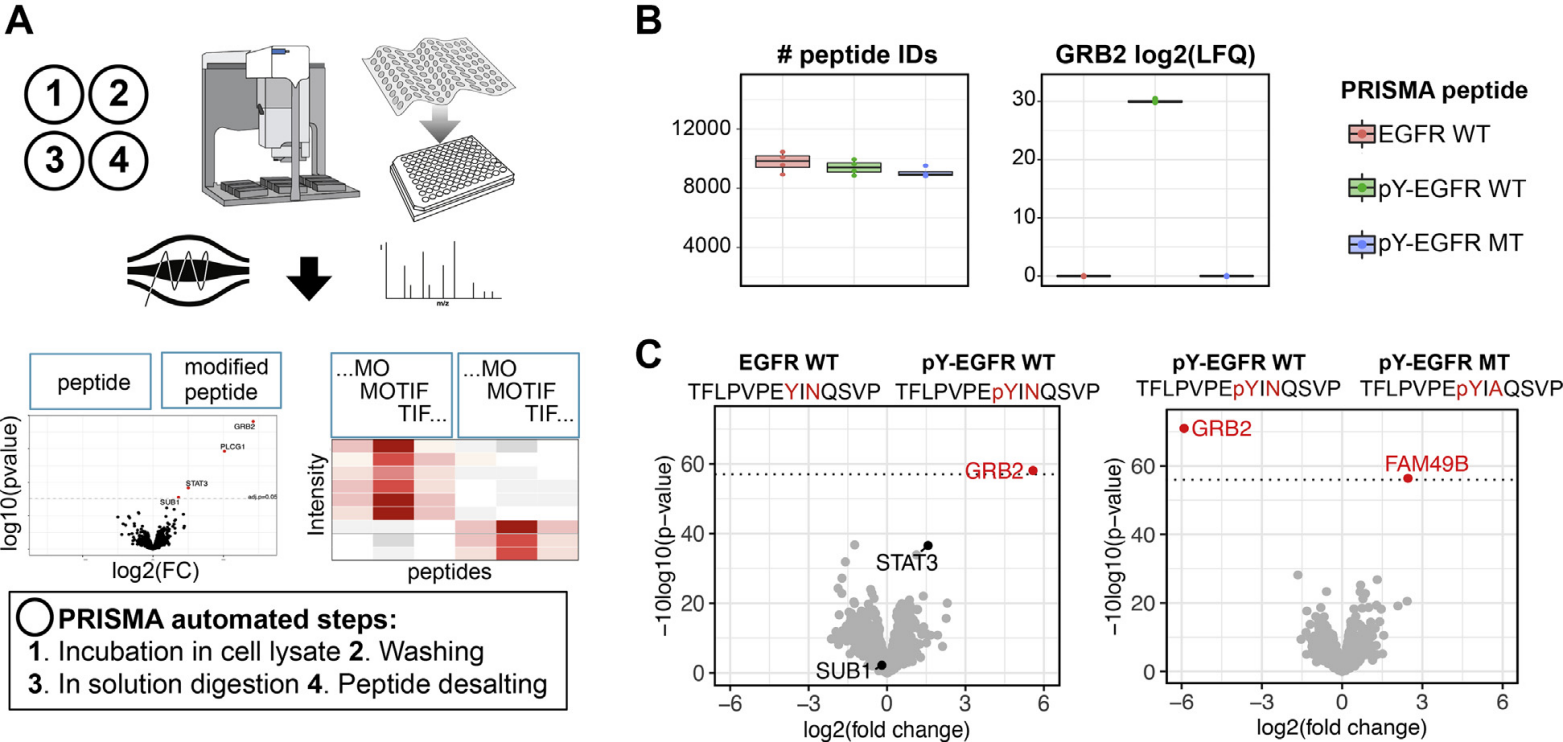
**Automation of the PRISMA workflow**. *A*, automated PRISMA workflow on an Agilent Bravo system. *B*, number of identified peptides and GRB2 LFQ intensity across EGFR WT, EGFR phosphorylated (pY1092), and EGFR phosphorylated mutated (pY1092, N1094>A) peptides. *C*, pairwise comparisons were performed with a moderated two-sample *t* test. Volcano plots depict fold changes of detected proteins plotted against their *p*-value. Significance cut-off (0.05 FDR) is indicated with a *dotted line*. Processed data are available in supplemental Table S8. FDR, false discovery rate; LFQ, label-free quantification; PRISMA, protein interaction screen on a peptide matrix.

To further increase sample throughput and to streamline the PRISMA workflow, we implemented the experimental procedure on an Agilent Bravo liquid-handling platform. Quadruplicates of three different EGFR PRISMA peptide spots were prepared in a 96-well plate and conditioned, incubated, washed, digested, and desalted in an automated procedure (Fig. 5*A*). In this mode, individual peptide spots are incubated and washed separately from each other in individual wells, as compared with the manual workflow in which the entire membrane was incubated with cell lysates and washed before punching out of individual peptide spots. Technical replicates for the automated procedure had an average Pearson correlation of 0.95, and the number of peptide and protein identifications was similar compared with numbers observed in the manual workflow (Fig. 5*B*). GRB2 was successfully identified as a differential interactor of the Y1092 phosphorylated EGFR peptide, demonstrating robustness for automated PRISMA (Fig. 5*C*, supplemental Table S8). STAT3 (FDR 0.099) did not pass the stringent significance cutoff of 0.05 FDR, but the differential interaction could be recovered by adjusting the FDR to 0.1. PLCG1 was not detected in the automated workflow while SUB1 did not show a significant change. Differences in results compared with the manual procedure may be related to the lack of constant cooling during automated workflows and the general variability of the PLCG1 signal that we also observed in optimization experiments (supplemental Fig. S1).

To further increase throughput, we also tested the EVOSEP ONE system (34) as an HPLC alternative to the EASY-nLC system with reduced run-to-run overhead time for the analysis of PRISMA samples (20 min linear gradient). The expected differential interactor GRB2 was successfully identified in both setups, although numbers of total protein identifications were approximately 20% lower in the EVOSEP setup (supplemental Fig. S5, supplemental Table S9). In summary, these results demonstrate feasibility of automation of PRISMA and that the throughput can be increased to the analysis of 60 samples per 24 h of machine time. Accordingly, PRISMA can be operated as a semiautomated high-throughput screening mode to identify peptide–protein interactions.

## Discussion

Despite the functional importance of protein interactions mediated by IDRs, systematic and unbiased detection still remains a technical challenge. For many years, immune purification coupled to MS was the gold standard for protein-interaction studies. One drawback of this method is the requirement of specific antibodies against the endogenous protein or genetic introduction of an affinity tag. In addition, dynamic transient interactors are easily lost during the purification process. Proximity labeling with BioID or APEX is becoming increasingly popular and offers significant advantages for the detection of dynamic protein interactions (35, 36). However, these methods require time-consuming cloning steps and target cell/tissue delivery and do not provide information about which parts of the protein and potential PTMs are involved in PPIs. While the introduction of deletions or point mutations in the bait is possible, it is rather laborious and does not allow for systematic screening or incorporation of PTMs. PTM mimicry point mutations, such as S/T/Y>D as surrogate phosphorylation, may not mirror the biochemical functions of a PTM in all cases (37).

Motif-based protein interactions, which do not require the larger structural context of the whole protein, can be recapitulated with synthetic peptides. Peptide arrays thus permit systematic and unbiased screening of hundreds of peptides in parallel for protein interactions. Peptide arrays coupled to MS have recently been developed for protein interaction mapping, as well as studying the influence of PTMs and point mutations on interactions (18, 19, 20). The main advantages of the PRISMA method and cellulose peptide arrays in general are the high local density of peptides and the low cost of array production. High local peptide concentrations may counteract the dissociation of low-affinity, transient interactors by providing alternative binding sites in direct vicinity after dissociation of the weak interactor leading to a higher avidity in general.

Here, we tested three different applications of PRISMA (PTMs, point mutations and mapping of SLiMs) with an optimized experimental protocol. The universal workflow permitted detection of phosphorylation-dependent interactors of EGFR, JNK1, and p38 peptides or mutation-dependent interactions of SOS1, GLUT1, and EGFR peptides. In addition, we mapped the interactions of the mediator of transcription and the anaphase-promoting complex to distinct CRs of CEBPB and confirmed previously described arginine methylation–dependent interactions of CEBPB (19). Together, the data demonstrate that PRISMA is applicable to study cytoplasmic and nuclear protein interactions.

The optimized protocol supports completion of hundreds of peptide pull-downs in parallel in a single day and allows acquisition of highly reproducible peptide–protein interaction data in a high-throughput manner. So far, the PRISMA workflow required manual retrieval of individual peptide spots by punching out respective areas on the cellulose membrane after incubation with the protein extract. Our results show that the PRISMA protocol can be transferred to a microwell format with fully automated robotic liquid handling. Accordingly, PRISMA has the potential to systematically explore intrinsically disordered protein functions and unravel SLiM-based protein networks in signaling processes and diseases.

## Data availability

The mass spectrometry proteomics data and search results have been deposited to the ProteomeXchange Consortium *via* the PRIDE partner repository (23) with the dataset identifier PXD027265.

## Supplemental data

This article contains supplemental data.

## Conflict of interest

The authors declare no competing interests.

## Supplemental data

Supplemental Experimental Procedures
