# Supplementary material for: A Universal Peptide Matrix Interactomics Approach to Disclose Motif-Dependent Protein Binding
Source: Mol Cell Proteomics. 2021 Aug 13;20:100135. doi: 10.1016/j.mcpro.2021.100135 (PMC8453223; doi:10.1016/j.mcpro.2021.100135)
Supplement: Supplemental Experimental Procedures [file mmc1.pdf]

**Supplemental Experimental Procedures**

**Universal PRISMA method**

**Buffers**

Modified RIPA buffer (store at 4°C):

50mM HEPES pH 7.6 at 4°C,
150mM NaCl,
1mM EGTA,
1mM MgCl2,
10% glycerol,
0.5% Nonidet P-40,
0.05% SDS
0.25% sodium deoxycholate
supplement before use with protease inhibitors (cOmplete™ Mini, Roche) and universal nuclease (Pierce, ThermoFisher)

PRISMA washing buffer (store at 4°C):

50mM HEPES pH 7.6 at 4°C,
150mM NaCl,
1mM EGTA,
1mM MgCl2,
10% glycerol

Denaturation buffer (DB) (store aliquots at -20°C or prepare fresh):

6M urea,
2M thiourea,
10mM HEPES

Reagents

10mM tris (2-carboxyethyl) phosphine (TCEP)
55mM 2-Chloroacetamide (CAA)
50mM ammoniumhydrogencarbonat (ABC)
Acetonitrile (ACN) hypergrade for MS
Formic acid (FA)
Lys-C (Wako, Japan)
Modified trypsin, sequencing grade (Promega, WI, USA)
Methanol MS grade
3M™ C18 Empore™ disks for STAGE tips [22]

1. Cell lysis with modified RIPA buffer

We recommend to use 100 µl lysis buffer for each 15 cm dish (80% confluence). Supplement lysis buffer just before use with protease inhibitors and universal nuclease for cell lysis (2 µl nuclease / 80% confluent 15 cm dish.)

- aspirate media and wash once on plate with ice cold PBS
- harvest cells by scraping in ice cold PBS
- collect cells by centrifugation at 1000g, 4°C, 5 min
- wash cells once more in ice cold PBS
- resuspend cell pellet in chilled lysis buffer supplemented with protease inhibitors and nuclease
- vortex 2 s at medium speed
- let sit on ice for 30 min
- vortex again 2 s at medium speed
- centrifuge at 18,000g, 4°C, 10 min
- transfer supernatant to a pre-chilled tube

- prepare aliquots and snap freeze extracts in liquid nitrogen
- keep a small aliquot for measuring protein concentration

## 2. PRISMA pull downs

For washing and incubation steps, a plastic box or cell culture dish of appropriate size can be used. The membrane should be covered with liquid at all times. To cover a membrane of 8 cm x 8 cm, typically ≥ 5 ml of protein extract at ≥ 4 mg/ml are needed. Washing and incubation steps are performed at 4°C on an orbital shaker.

- thaw protein extract on ice. Centrifuge extract at 18,000g, 4°C, 10 min
- transfer supernatant into new tube and dilute dropwise to desired concentration (≥ 4 mg/ml) with lysis buffer + protease inhibitors.
- in the meantime, place peptide membrane in incubation box and hydrate in wash buffer for 15 min at RT
- discard wash buffer
- carefully add protein extract to incubation box
- place box on ice
- incubate for 20 min while shaking
- remove protein extract
- wash membrane 3x for 5 min with ice cold washing buffer while shaking (use > 30 ml buffer for each washing step)
- place membrane on glass plate or other inert surface to dry (takes approximately 1 h)
- meanwhile, prepare 20 µl of DB in each well of a 96 well plate
- punch peptide membrane spots (2 mm biopsy punch, Stiefel) and place the spot in the correct well. Keep plate on ice until all samples are ready
- plates can be frozen at -80°C until digestion or digested immediately
- suggested QC: ponceau staining of punched out membrane to verify that all spots have been punched out correctly

## 3. In solution digestion

- reduce each sample by adding 2 µl of 10mM TCEP
- incubate for 30 min at RT
- alkylate each sample with 2 µl 55mM of CAA
- incubate for 45 min at RT
- dilute each samples with 100 µl 50mM ABC to a urea concentration < 2M
- add 0.5µg trypsin (1 µl) and 0.5 mAU LysC (1 µl) per sample
- digest samples overnight (16 h) at RT
- acidify each sample by adding 4 µl of 25% TFA

## 4. Peptide cleanup

We recommend peptide cleanup using the STAGE (STop And Go Extraction) TIPS Desalting procedure [22], but also other peptide cleanup methods may be used.

- assemble two disks of C18 material in a 200 µl pipet tip for each sample. Place STAGE TIPS into centrifuge with adapters
- STAGE TIPS conditioning (2500g, 2 min for each step):
  - 1 x 50 µl MeOH
  - 1 x 100 µl 50% ACN/0.1% FA
  - 1 x 100 µl 1% FA
- load acidified samples (1500g, 4 min)
- wash samples (2500g, 2min for each step)
  - 2x 100 µl 0.1% TFA
  - 1 x100 µl 1% FA

- elute samples (1500g, 3 min for each step)
  1x 50 µl 50% ACN/0.1% FA
- speedvac to dryness

## Supplemental figure legends

**Figure S1: Optimization of PRISMA experimental parameters with EGFR peptides, related to Figure 1**

**A:** STAT3 or PLCG1 LFQ intensity across EGFR WT, EGFR phosphorylated (pY1092) and EGFR phosphorylated mutated (pY1092, N1094 -> A) peptides dependent on protein lysate concentration (n = 4), incubation time (n = 4) and LC gradient length (n = 3). **B**: Influence of washing temperature on number of peptide identifications, GRB2, STAT3 and PLCG1 LFQ intensities. After incubation with cell lysates at 4°C (4mg/ml, 45 min), samples were washed at 4˚C or room temperature (n=3). **A-B**: Boxplots display median of non-zero measurements.

**Figure S2: Enabling the match between run (MBR) option of MaxQuant influences PRISMA results, related to Figure 2 and Figure 1**

**A**: Pairwise comparison of mutated (MT) vs. wildtype (WT) peptides of WIP with or without using the match between run (MBR) option of MaxQuant. Significance threshold of 0.05 FDR is indicated with a dotted line (LFQ analysis, comparison of 4 replicates with moderated 2sample t-test). **B:** Venn diagrams show overlap of significant proteins without (red circles) or with (blue circles) using MBR in PRISMA experiments comparing wild type and mutant peptides of WIP1, GLUT1 or SOS1. The Venn diagram of SOS1 shows only SH3 domain containing proteins. **C:** GRB2 LFQ intensity across EGFR WT, EGFR phosphorylated (Y1092) and EGFR phosphorylated mutated (Y1092, N1094 -> A) peptides dependent on protein lysate concentration (n = 4). Data was analyzed with or without MBR.

**Figure S3: Impact of cell lysate type on PRISMA results, related to Figure 3, Table S3, Table S4**

Pairwise comparison of PRISMA pulldowns of unmodified and phosphorylated JNK1 and p38α peptides. Modified RIPA or commercial nuclear extract from HeLa cells were used as indicated. **A**: Volcano plots highlight differential interactors of phosphorylated peptides **B**: GO term enrichment of differential interactors **C**: Venn diagrams show overlap of differential interactors. SH2 domain containing interactors are highlighted in red. **D**: Pearson correlation heatmap of log2 LFQ intensities in all experiments.

**Figure S4: PRISMA CEBPB samples cluster together based on their interactome, related to Figure 4, Table S4**

Correlation matrix (A) and principal component analysis (B) of PRISMA pulldowns with unmodified CEBPB peptides covering CR2 and CR7.

**Figure S5: Comparison of EASY-nLC and Evosep chromatography systems for PRISMA. , related to Figure 1B, Table S2, Table S6**

For the EASY-nLC system, a 20 min linear gradient (45 min total method length) was used. For the evosep system, the preset 60 samples per day method (20 min) was used. Bar graphs represent number of identified peptides and GRB2 LFQ intensity across EGFR WT, EGFR phosphorylated (pY1092) and EGFR phosphorylated mutated (pY1092, N1094> A) peptides.

**Supplemental table legends**

**Table S1: List of peptides screened for protein interactions with the optimized PRISMA method.**

**Table S2: PRISMA optimization of protein lysate concentration.**
PRISMA was performed with EGFR, EGFR phosphorylated (pY1092) and EGFR phosphorylated mutated (pY1092, N1094A) peptides. Different RIPA lysate protein lysate concentrations (1, 2, 4, 8 mg/ml) were tested in quadruplicates. Table reports Protein IDs, number of peptide identifications, LFQ intensities and raw intensities per sample. MS data was analyzed with MaxQuant without match between runs.

**Table S3: PRISMA optimization of incubation time.**
PRISMA was performed with EGFR, EGFR phosphorylated (pY1092) and EGFR phosphorylated mutated (pY1092, N1094A) peptides. Different incubation times (10, 20, 45, 90 min) were tested in quadruplicates. Table reports Protein IDs, number of peptide identifications, LFQ intensities and raw intensities per sample. MS data was analyzed with MaxQuant without match between runs.

**Table S4: PRISMA optimization of gradient length.**
PRISMA was performed with EGFR, EGFR phosphorylated (pY1092) and EGFR phosphorylated mutated (pY1092, N1094A) peptides. Different LC gradient lengths (10, 20, 45) were tested in triplicates. Table reports Protein IDs, number of peptide identifications, LFQ intensities and raw intensities per sample. MS data was analyzed with MaxQuant without match between runs.

**Table S5: PRISMA optimization of washing temperature.**
PRISMA was performed with EGFR, EGFR phosphorylated (pY1092) and EGFR phosphorylated mutated (pY1092, N1094A) peptides. Different washing temperatures (RT, 4°C) were tested in triplicates. Table reports Protein IDs, number of peptide identifications, LFQ intensities and raw intensities per sample. MS data was analyzed with MaxQuant without match between runs.

**Table S6: Results of optimized PRISMA with modified RIPA lysis buffer.**
Peptides listed in Table S1 were screened for protein interactions with the optimized PRISMA method in quadruplicates. MS data was analyzed with MaxQuant without match between runs. Identifications were filtered for at least 3 valid values per group and pairwise comparisons were performed with a moderated t-test after sample wise imputation of missing values.

**Table S7: Results of optimized PRISMA with nuclear extract.**

Peptides listed in Table S1 were screened for protein interactions with the optimized PRISMA method in quadruplicates. MS data was analyzed with MaxQuant without match between runs. Identifications were filtered for at least 3 valid values per group and pairwise comparisons were performed with a moderated t-test after sample wise imputation of missing values.

**Table S8: Results of automated PRISMA on a BRAVO liquid handling platform.**

Incubation with protein extract, washing, digestion and desalting were performed on an AssayMap BRAVO liquid handling platform (Agilent). EGFR, EGFR phosphorylated (pY1092) and EGFR phosphorylated mutated (pY1092, N1094A) peptides were analyzed in quadruplicates. MS data was analyzed with MaxQuant without match between runs. Identifications were filtered for at least 3 valid values per group and pairwise comparisons were performed with a moderated t-test after sample wise imputation of missing values.

**Table S9: Results of PRISMA measured with the EVOSEP LC system.**

PRISMA was performed with EGFR, EGFR phosphorylated and EGFR phosphorylated mutated peptides. Samples were analyzed with the EVOSEP LC system (60 samples a day method) in quadruplicates. Table reports Protein IDs, number of peptide identifications, LFQ intensities and raw intensities per sample. MS data was analyzed with MaxQuant without match between runs.

# Supplemental figures

## Figure S1

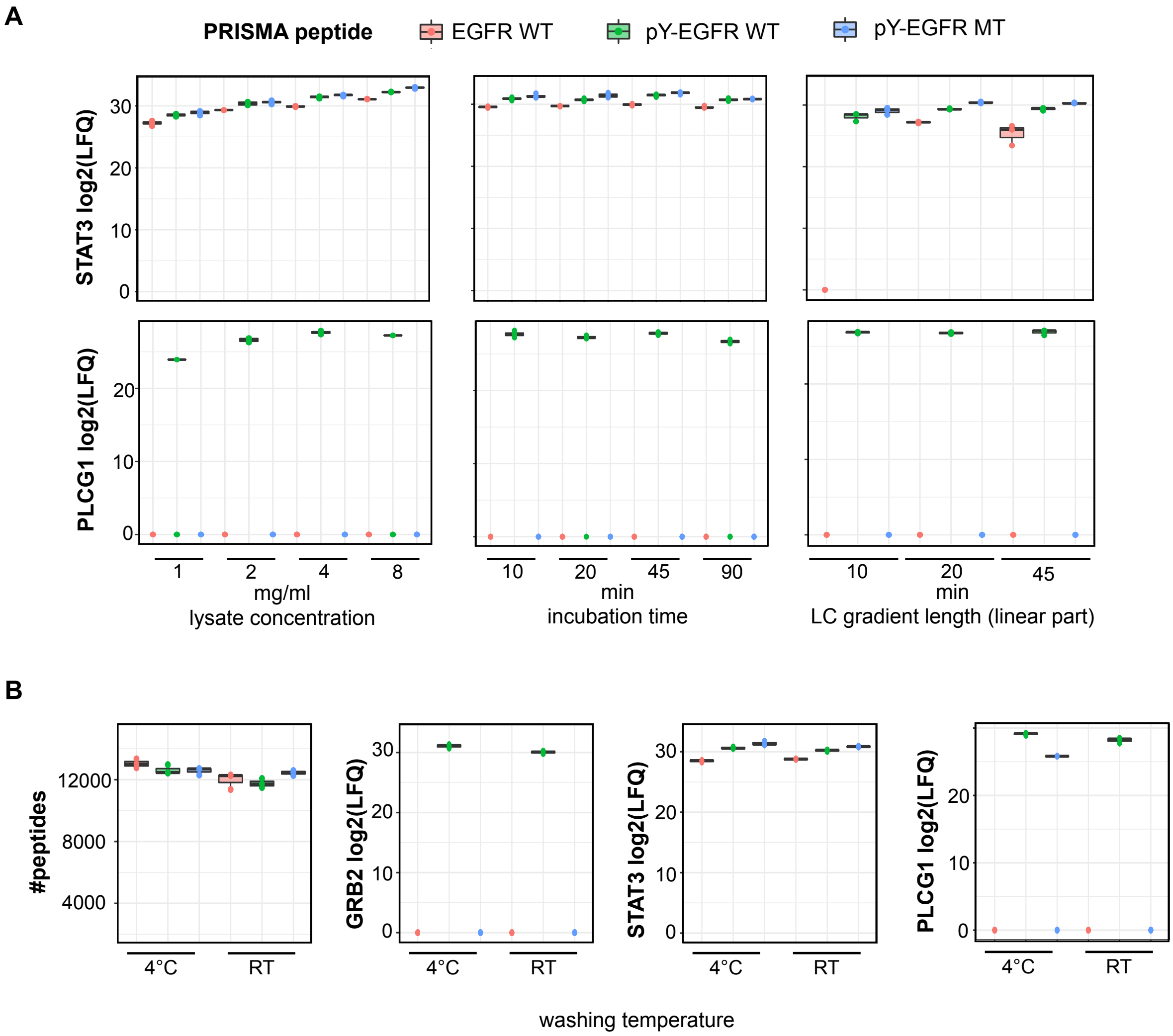

## Figure S2

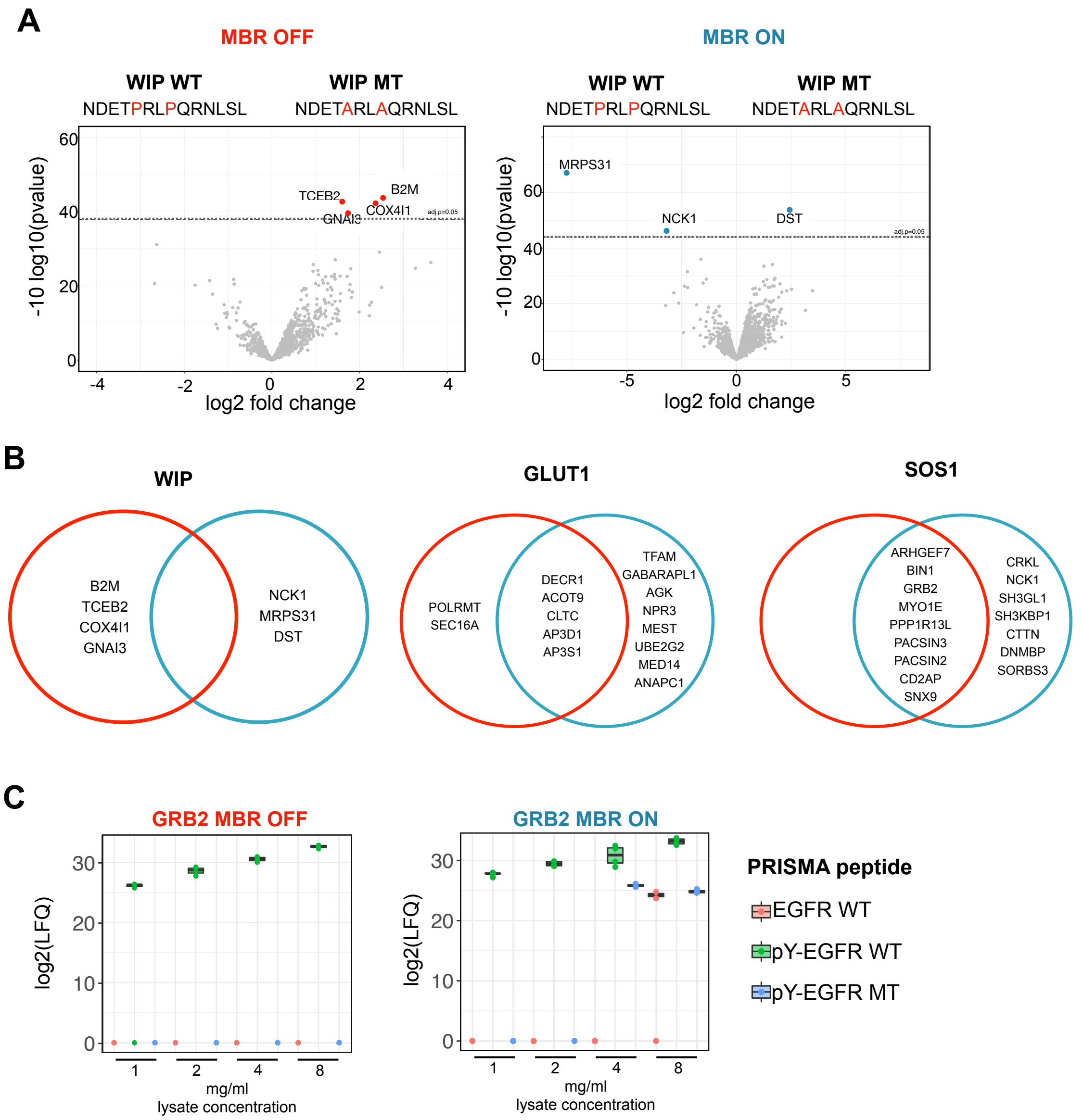
A
MBR OFF
MBR ON
WIP WT
NDETPRLPQRNLSL
WIP MT
NDETARLAQRNLSL
-10 log10(pvalue)
log2 fold change
TCEB2
B2M
COX4I1
GNAI3
MRPS31
NCK1
DST
adj.p=0.05
B
WIP
B2M
TCEB2
COX4I1
GNAI3
NCK1
MRPS31
DST
GLUT1
POLRMT
SEC16A
DECR1
ACOT9
CLTC
AP3D1
AP3S1
TFAM
GABARAPL1
AGK
NPR3
MEST
UBE2G2
MED14
ANAPC1
SOS1
ARHGEF7
BIN1
GRB2
MYO1E
PPP1R13L
PACSIN3
PACSIN2
CD2AP
SNX9
CRKL
NCK1
SH3GL1
SH3KBP1
CTTN
DNMBP
SORBS3
C
GRB2 MBR OFF
GRB2 MBR ON
log2(LFQ)
mg/ml
lysate concentration
PRISMA peptide
EGFR WT
pY-EGFR WT
pY-EGFR MT

**Figure S3**

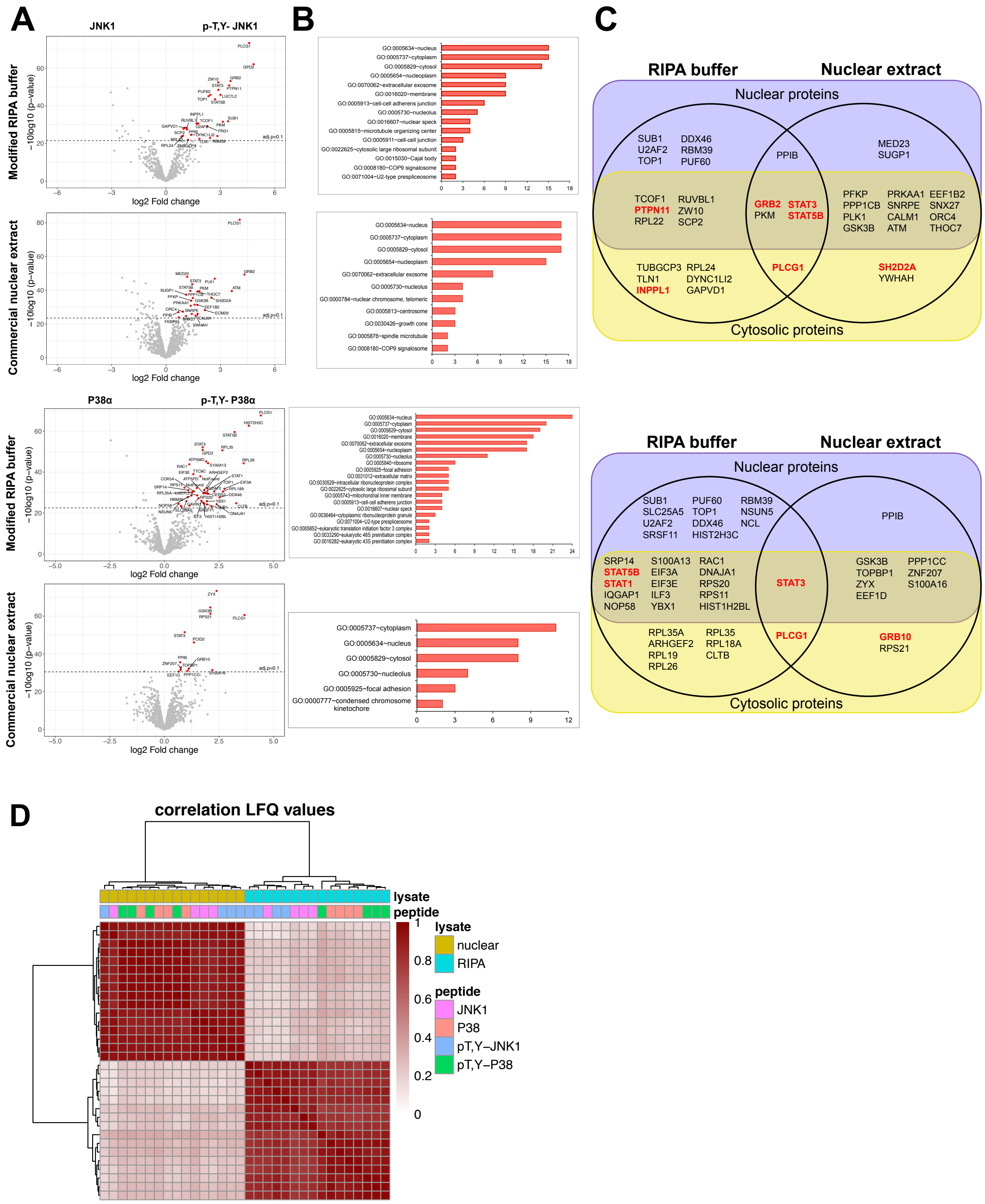

A
JNK1
p-T,Y- JNK1
Modified RIPA buffer
Commercial nuclear extract
log2 Fold change
−10log10 (p−value)
P38α
p-T,Y- P38α
B
GO:0005634~nucleus
GO:0005737~cytoplasm
GO:0005829~cytosol
GO:0005654~nucleoplasm
GO:0070062~extracellular exosome
GO:0016020~membrane
GO:0005913~cell-cell adherens junction
GO:0005730~nucleolus
GO:0016607~nuclear speck
GO:0005815~microtubule organizing center
GO:0005911~cell-cell junction
GO:0022625~cytosolic large ribosomal subunit
GO:0015030~Cajal body
GO:0008180~COP9 signalosome
GO:0071004~U2-type prespliceosome
GO:0000784~nuclear chromosome, telomeric
GO:0005813~centrosome
GO:0030426~growth cone
GO:0005876~spindle microtubule
GO:0005925~focal adhesion
GO:0000777~condensed chromosome kinetochore
C
RIPA buffer
Nuclear extract
Nuclear proteins
SUB1 DDX46
U2AF2 RBM39
TOP1 PUF60
PPIB
MED23
SUGP1
TCOF1 RUVBL1
PTPN11 ZW10
RPL22 SCP2
GRB2 STAT3
PKM STAT5B
PFKP PRKAA1 EEF1B2
PPP1CB SNRPE SNX27
PLK1 CALM1 ORC4
GSK3B ATM THOC7
TUBGCP3 RPL24
TLN1 DYNC1LI2
INPPL1 GAPVD1
PLCG1
SH2D2A
YWHAH
Cytosolic proteins
RIPA buffer
Nuclear extract
Nuclear proteins
SUB1 PUF60 RBM39
SLC25A5 TOP1 NSUN5
U2AF2 DDX46 NCL
SRSF11 HIST2H3C
PPIB
SRP14 S100A13 RAC1
STAT5B EIF3A DNAJA1
STAT1 EIF3E RPS20
IQGAP1 ILF3 RPS11
NOP58 YBX1 HIST1H2BL
STAT3
GSK3B PPP1CC
TOPBP1 ZNF207
ZYX S100A16
EEF1D
RPL35A RPL35
ARHGEF2 RPL18A
RPL19 CLTB
RPL26
PLCG1
GRB10
RPS21
Cytosolic proteins
D
correlation LFQ values
lysate
peptide
lysate
nuclear
RIPA
peptide
JNK1
P38
pT,Y−JNK1
pT,Y−P38

## Figure S4

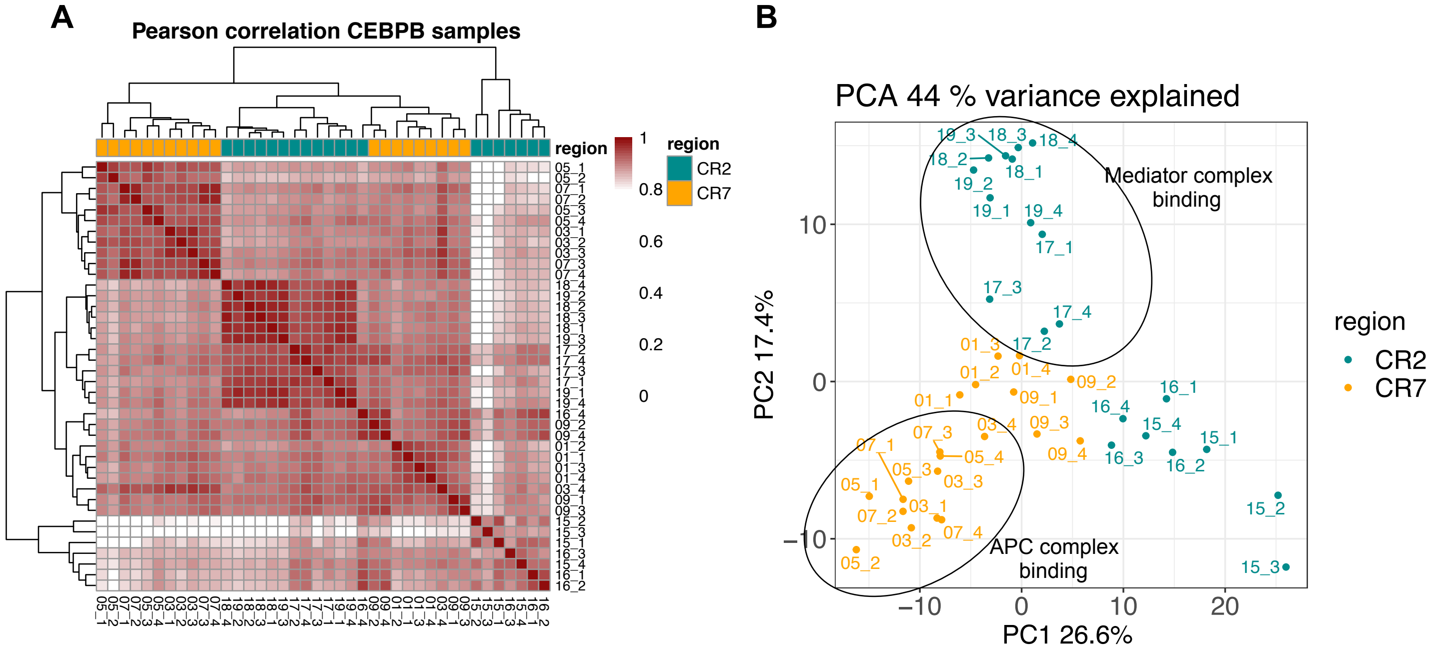
A
Pearson correlation CEBPB samples
region
CR2
CR7
1
0.8
0.6
0.4
0.2
0
B
PCA 44 % variance explained
Mediator complex binding
APC complex binding
PC2 17.4%
PC1 26.6%
region
CR2
CR7

## Figure S5

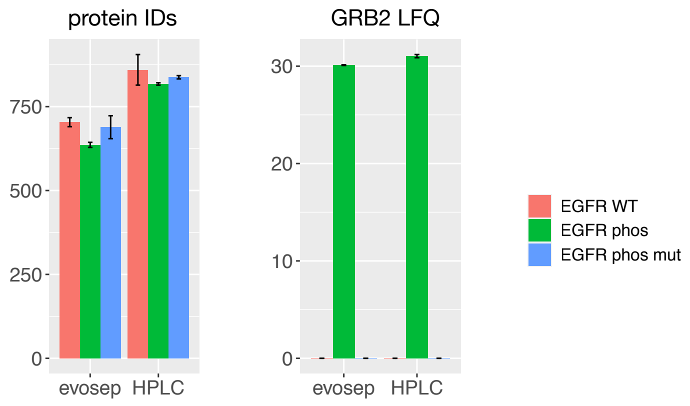

protein IDs
750
500
250
0
evosep
HPLC
GRB2 LFQ
30
20
10
0
evosep
HPLC
EGFR WT
EGFR phos
EGFR phos mut
